# Impact of Lithium-Ion Battery State of Charge on In Situ QAM-Based Power Line Communication

**DOI:** 10.3390/s22166144

**Published:** 2022-08-17

**Authors:** Mahyar J. Koshkouei, Erik Kampert, Andrew D. Moore, Matthew D. Higgins

**Affiliations:** National Automotive Innovation Centre, WMG, University of Warwick, Coventry CV4 7AL, UK

**Keywords:** power line communication, state of charge, quadrature amplitude modulation, impedance, scattering parameters, error vector magnitude, lithium-ion, smart cell, battery

## Abstract

Power line communication within a lithium-ion battery allows for high fidelity sensor data to be transferred between sensor nodes of each instrumented cell within the battery pack to an external battery management system. In this paper, the changing characteristics of the lithium-ion cell at various states of charge are measured, analysed, and compared to understand their effectiveness on the communication channel of a power line communication system for carrier frequencies of 10 MHz to 6 GHz. Moreover, the use of quadrature amplitude modulation (QAM) is investigated to determine its effectiveness as a state-of-the-art modulation method for the same carrier frequency range. The overall results indicate that certain carrier frequencies and QAM orders may not be suitable for the in situ battery pack power line communication due to changes in battery impedance with certain lithium-ion cell states of charge, which cause an increase in error vector magnitude, bit error ratio, and symbol error ratio. Recommendations and trends on the impact of these changing characteristics based upon empirical results are also presented in this paper.

## 1. Introduction

Limiting the effects of climate change requires immediate action in reducing emissions of greenhouse gasses such as carbon dioxide. The use of renewable energy resources is viewed as a crucial step to achieve this; hence, there is great momentum to research and incorporate such technology within infrastructure. The success of renewable resources heavily depends on the performance of renewable energy storage systems. Such systems include portable electronics, battery electric vehicles (BEVs), and large-scale energy storage [1].

BEVs have a reduced impact on climate change when powered from renewable resources in comparison to traditional internal combustion engine vehicles. However, there are significant limitations of using and adopting existing BEVs. Such restrictions include a limited driving range on a single charge, long charging time, and perceived comparative high purchase and running costs in comparison with internal combustion engine vehicles [2,3].

An important disadvantage of some renewable energies is their dependence on weather, such as the photovoltaic cell and the wind turbine. Therefore, large-scale energy storage systems are required to store power from these intermittent sources of energy for later use as demand requires [4].

### 1.1. Battery Management and Safety

The lithium-ion (Li-ion) battery is typically selected for use within energy storage systems due to its relative high power density, high energy density, long cycle life and high efficiency in comparison to other battery chemistries [5]. Nevertheless, a Li-ion battery is damaged by overcharging and deep discharging, by overcurrent during charging and discharging, and by internal and external short circuits. Hence, a battery management system (BMS) is required to monitor battery status and mitigate such failures [6]. The BMS performs real-time monitoring of the battery’s state of charge (SoC) to provide such mitigations [7]. An estimation of the state of health (SoH) of the Li-ion battery may also be used to measure ageing, such as in capacity and performance, which worsens over time and reduces the maximum power output of the battery [8]. These safety features provided by the BMS for Li-ion batteries are required; however, they restrict opportunities in performance optimisation of Li-ion batteries. Improved characterisation of the Li-ion cell may reduce the impact of safety strategies on the performance of Li-ion cells by providing closer tracking of more cell characteristics, such as SoC and SoH, enabling greater BMS intelligence use when assessing cell risk profile.

Further research is ongoing and required to improve Li-ion batteries, including safety, efficiency, power delivery, and also reducing and mitigating for battery ageing. Research is ongoing into the characterisation of the Li-ion cell, providing further techniques in improving the performance of the BMS and the Li-ion battery. The typical BMS normally provides certain estimations on the characteristics of the battery. Such techniques for SoC and SoH estimations have been discussed in [9], which include battery modelling, Kalman filters, intelligent algorithms, and neural network algorithms.

### 1.2. Cell Instrumentation and Sensing

Some research has been conducted to obtain applicable techniques in fast charging Li-ion cells [10]. Such techniques include the instrumentation of the Li-ion cell for measuring the core temperature of the cell that may then be used for accurate cell modelling and analysis. A thin optical fibre was used for temperature sensing and placed on the top and bottom surfaces of a Li-ion pouch cell, and also in between two other pouch cells [11]. The C-rate is the rate at which a battery is charged or discharged relative to its capacity. During a 1 C-rate discharge cycle (i.e., the cell is fully discharged in one hour), temperature hot spots on the surface of the pouch cells were found with temperature variations of up to 8 °C. In this direction, a Rayleigh scattering based optical fibre has been utilised to improve the temperature sensing accuracy of the optical fibre placed on the surface of a Li-ion pouch cell [12]. In this work, it was found that during a 5 C-rate discharge at an ambient temperature of 10 °C a maximum in-plane temperature variation of 11.8 °C was recorded with the optical fibre-based temperature sensing method. In comparison, the traditional thermocouple-based temperature sensing method recorded a maximum in-plane temperature variation of only 2.9 °C, which was a vast underestimation in surface temperature, and demonstrated a comparative insensitivity to hot spots. In another research work, an optical fibre with Bragg gratings embedded within a Li-ion cylindrical cell was used to measure the core temperature [13]. It was demonstrated that the core temperature and the surface temperature were different during mild cell cycling (i.e., repeated charging and discharging), which indicates candidacy for improved cell characterisation. A review on further optical fibre temperature sensing techniques can be found in [14].

### 1.3. Smart Cells

For these stated in situ sensing techniques, an external system is necessary to collect and process the acquired data for each Li-ion cell. For large-scale batteries that incorporate many individual cells, such as those used within BEVs, a direct connection of sensors from each cell to an external system, such as the BMS using a wire harness, is an overhead within a battery, potentially impairing performance, energy density and robustness in a harsh environment, requiring extra space, higher wire termination count and additional weight. Embedding some BMS functionality within an instrumented Li-ion cell is known as the ‘cell management system’ and is typically named ‘smart cells’. The advantages of smart cells include higher operational flexibility, fault tolerance, safety management, equalisation, and life management and history [15]. A decentralised system of three smart cells, each a Li-ion cell with an embedded system, has been designed and tested without the use of an external BMS [16] including the smart cell performing estimations of cell voltages during operation. For large-scale batteries, a communication system would provide additional benefits as was recommended, following the distributed cell management experiments.

A self-balancing system of eight smart cells has also been designed and demonstrated using controller area network (CAN bus) communication between each smart cell for sharing cell voltage and SoC information [17]. A two-wire power communication bus for smart Li-ion cells was presented in [18], where the embedded systems within cells are additionally powered from the communication bus.

Ongoing research attempts to remove the need for any additional wire harness within a battery pack for cell-to-cell and cell-to-BMS communication. These techniques include the use of wireless communication and power line communication (PLC), which may be utilised to communicate with other smart cells as part of a mesh network, or with a BMS. A modular BMS with a wireless communication system has been designed in [19] with a particular focus on low power consumption. A wireless communication system embedded within a single 18650-model cylindrical Li-ion cell was simulated, built and demonstrated [20]. In this study, a wireless communication range of up to 10 m has been demonstrated and the use of an SMA connector and also an SMA connector with a wire loop antenna was tested. Further research is required to determine the effectiveness of in situ cell wireless communication systems within large-scale batteries.

### 1.4. In Situ Battery Power Line Communication

PLC uses the power terminals of the battery, such as the bus bar, as a communication medium. Similarly to the wireless communication research already described, no additional wire harness is required within the battery for smart cell PLC communication. An instrumented smart cell system utilising PLC communication with four cylindrical 21700 model Li-ion cells in the 2S2P arrangement was presented in [21]. In this study, bi-directional communication was demonstrated using a PLC modem capable of carrier frequencies of 1 MHz to 5 GHz with a data rate up to 500 kbps during drive cycle experimentation and cell cycling. This PLC system was tested at cell SoCs of 100%, 80%, 50%, 30%, which demonstrated a peak temperature increase when the cells were at 30% SoC. In [22] an instrumented Li-ion pouch cell was tested with an in situ PLC system using a carrier frequency of 6.5 MHz. It has been demonstrated that PLC had no effect on the electrochemical performance of the cell. In another work, four fully charged 18650-model Li-ion cells have been tested within a PLC system using three combinations of capacitive coupling techniques with two different cell brands, measuring the system impedance and received signal strength using a vector network analyser (VNA) and spectrum analyser, respectively [23]. In addition, carrier frequencies of 1 MHz to 3 GHz were tested, and it was found that the maximum signal attenuation was 65 dB. In [24], the authors of this work simulated the use of quadrature amplitude modulation (QAM) with PLC on two Li-ion cells in series and also two Li-ion cells in parallel. A Li-ion battery model of cell impedance was used, and carrier frequencies of 1 MHz to 200 MHz were tested for their effectiveness within the system. It was found that both the battery configuration and the carrier frequency had an effect on the communication performance. In [25], a VNA was used to perform measurements on the scattering parameter S21 for a single Li-ion cell in a shunt-through configuration for frequencies of 1 kHz to 300 MHz. The cell impedance and the insertion loss were calculated using the recorded S21 transmission coefficient, and it was determined that the cell impedance had reached 40Ω at 300 MHz.

### 1.5. Paper Contributions and Organisation

In this paper, the effects of Li-ion cell SoC on an in situ PLC system are evaluated for carrier frequencies of 10 MHz to 6 GHz. S-parameter S21 analysis on a single 18650-model Li-ion cell in a series-through configuration is performed using a VNA to characterise the Li-ion cell as a communication channel. A vector signal transceiver (VST) is used to perform actual tests of in situ Li-ion PLC using QAM. These tests are carried out when the Li-ion cell is discharged to 95%, 80%, 60%, 40%, 20%, 5% SoC. The performance of this PLC system is quantified by quality of signal parameters obtained using the VST, which are error vector magnitude (EVM), bit error ratio (BER), and symbol error ratio (SER). Comparisons are made between the changes of the PLC performance with the different SoCs tested. Furthermore, QAM is used to demonstrate the efficient use of the communication channel to achieve higher data rates.

The key contributions of this work are as follows:

1. Measurement and analysis of a Li-ion cell as a PLC channel with QAM at various carrier frequencies between 10 MHz to 6 GHz, with an emphasis on how the forward transmission coefficient of this channel changes when the Li-ion cell is discharged to 95%, 80%, 60%, 40%, 20%, 5% SoC.
2. Measurement and analysis of communication performance using real PLC tests through a Li-ion cell using QAM.
3. Recommendations for prospective in situ PLC systems based upon empirical evidence, such as appropriate carrier frequencies and QAM orders.

This paper is organised as follows: Section 2 addresses the methodology used to carry out the analysis of the Li-ion communication channel. Section 3 presents the experimental results and their analysis. Section 4 provides conclusions and recommendations based upon the acquired empirical evidence. Detailed communication error figures are presented in Appendix A.

## 2. Methodology

In this section, experimental details on the methodology and equipment used in this study are presented.

### 2.1. Channel Analysis

To determine the effectiveness of a Li-ion cell as an effective communication channel for power line communication, an analysis of the channel’s S21 transmission coefficient needs to be performed. The transmission coefficient of the PLC channel allows for the analysis of signal attenuation and phase shift imposed onto the signal by the channel. Measuring the transmission coefficient of the Li-ion cell at various carrier frequencies enables finding the most appropriate parameters of the PLC system, including carrier frequency, filter requirements, and QAM order. Such parameters will also take into account the changes in transmission coefficient caused by alterations in the Li-ion cell SoC. Based on the authors’ preliminary work [26], it is expected that a change in impedance, and hence the channel transmission coefficient, due to the SoC of the Li-ion cell, may lead to variations in attenuation and phase shift, which as a consequence, influences data integrity as measured with EVM, BER, and SER. Hence, for the most effective PLC parameters, the impedance magnitude and phase shift must remain the same throughout all SoCs tested.

The S-parameter S21 represents the forward transmission coefficient of the Li-ion cell as a communication channel. In this experiment, the VNA is configured for an S21 measurement. S21 is defined as the ratio of the wave transmitted by the VNA at port 1 to the measured wave at port 2. The S21 transmission coefficient can be used to determine other features of the communication channel, such as the impedance and insertion loss. As in [25], given that the characteristic impedance $Z_{0}$ of the VNA is 50 Ω, the impedance of the cell $Z_{cell}$ is calculated as follows

(1) $$Z_{cell}=\frac{Z_{0}}{2}\cdot \frac{S_{21}}{\left(1-S_{21}\right)}$$

Insertion loss IL is given by

(2) $$IL=20\cdot \log\left|S_{21}\right|$$

Further details of scattering parameters can be found in [27].

Using the impedance and the phase shift, it is possible to calculate the reactance and the internal resistance of the Li-ion under a test. The relationships between them can be expressed as

(3) $$\begin{array}{ll} Z & =\sqrt{R^{2}+X^{2}}\\ X & =Z\sin\phi \\ R & =Z\cos\phi \end{array}$$

where *Z*, *X* and *R*, indicate impedance, reactance, and resistance, respectively.

**Figure 1:**
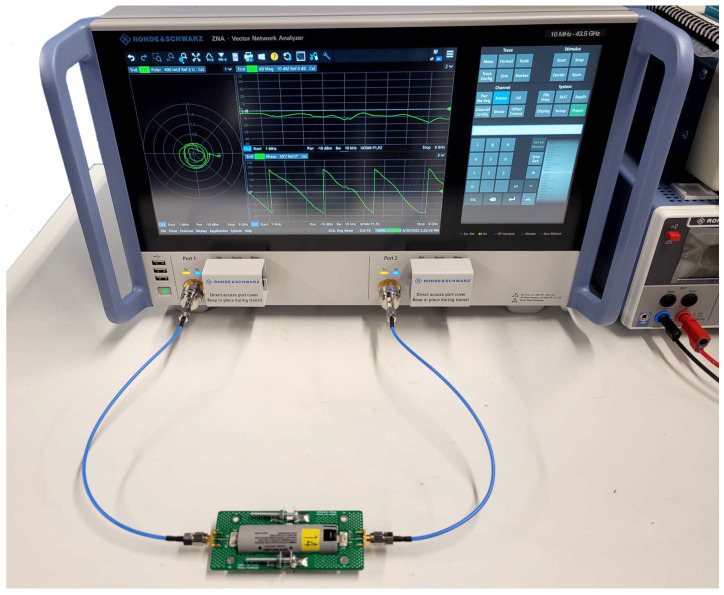
Photo of the Li-ion cell connected to the VNA. The Li-ion cell is clamped within the bespoke cell holder as described in Section 2.3.

**Figure 2:**
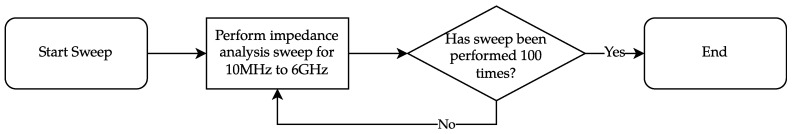
Flowchart of the specific experimental process for the VNA.

In this experimental work, a Rohde & Schwarz (R&S, Coventry, UK) ZNA43 vector network analyser shown in Figure 1 is used to perform S21 measurements of the PLC channel. The VNA is configured to perform a sweep of S21 measurements over the frequency range from 10 MHz to 6 GHz with a step of 1 MHz. This sweep is performed 100 times in order to reduce the impact of noise, interference, outliers after averaging, and ultimately improve the accuracy and confidence of the results obtained. The S21 data is then processed to determine the median for each frequency tested. The series of 100 sweeps are performed for each SoC tested, as shown in the experimental process in Figure 2.

The VNA is configured to record the S21 transmission coefficient using a preconfigured calibration profile performed before commencing the experiment. This profile utilises the R&S ZN-Z129 calibration kit, for a calibration profile spanning frequencies of 10 MHz to 40 GHz. This calibration profile allows for accurate S21 magnitude and phase measurements with the VNA. It is observed that the cell holder and the Li-ion cell have a longer signal path, i.e., electrical length, than the ZN-Z129 calibration kit. Thus, the phase compensation applied by the VNA using the ZN-Z129 calibration profile will be inadequate to effectively compensate for the phase shift caused by the longer signal path. Therefore, the measured phase shift will include a linear background value, which will be compensated for during data processing to obtain only the characteristics of Li-ion cell on the phase of the signal.

The VNA is connected to the cell holder such that the stimulus signal is output to the negative terminal of the Li-ion cell, as is described in Section 2.3. Moreover, the VNA is connected to the cell holder using a coaxial cable with 2.92 mm SMA connectors rated for frequencies up to 40 GHz. However, despite the 40 GHz rating of the VNA and the cable, this experiment will limit the highest frequency tested to 6 GHz, as this is the maximum frequency that can be tested using the VST, as explained in Section 2.2. This part of the experiment results in a series of plots that show alterations in Li-ion cell characteristics with frequency and SoC.

During this experiment, the S-parameters of the Li-ion cell will be its only varying property with changing SoC. In which case, the impedance characteristics of the experimental components, such as the cell holder, will not change. Therefore, using a baseline measurement of the Li-ion cell S-parameters will remove the characteristic transmission coefficient of these experimental components, thereby only presenting the change in S21 caused only by changes to the Li-ion cell SoC. Hence, a baseline of 95% SoC is selected, as it is the first measurement performed within this experiment. These results are used to identify changes in the transmission coefficient of the cell with SoC at various carrier frequencies. In this regard, measuring the absolute transmission coefficient of the Li-ion cell is not an objective of this research. These results include an S21 magnitude plot displaying the attenuation of the stimulus signal through the cell holder, and a plot displaying changes in phase shift, relative to their respective baselines at 95% SoC.

### 2.2. Communication Error Analysis

**Figure 3:**
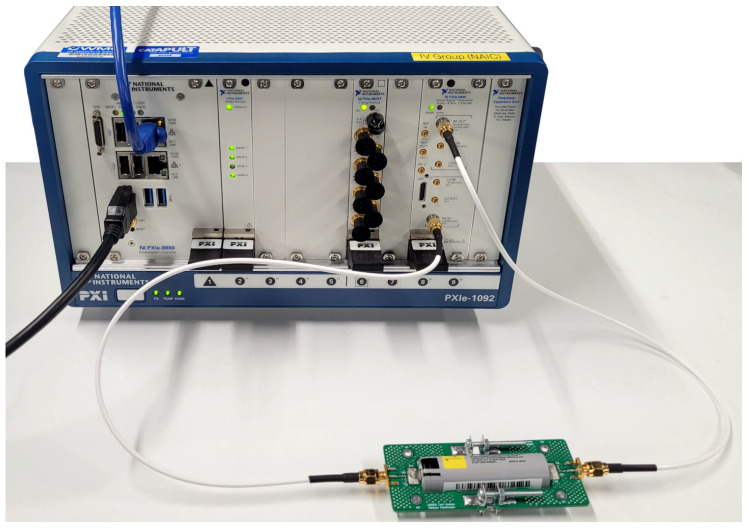
Photo of the Li-ion cell connected to the VST. The Li-ion cell is clamped within the bespoke cell holder as described in Section 2.3.

To determine the effectiveness of a Li-ion cell as a PLC channel using QAM, a VST is used. In this study, a NI PXIe-5840 VST and PXIe-8880 controller within a PXIe-1092 chassis, as shown in Figure 3, are used to transmit and to receive QAM symbols at carrier frequencies of 10 MHz to 6 GHz with a step of 50 MHz. In comparison to the S21 measurements, this increased frequency step is chosen to complete the experiment within a reasonable amount of time. QAM uses both amplitude shift keying (ASK) and phase shift keying (PSK) as modulation for enhanced utilisation of the transmission channel. The QAM modulation order is defined as 2 to the power of the number of transmitted bits per symbol, whereby, for instance, 16-QAM uses 4 bits of data in each symbol ($2^{4}=16$), allowing for 16 possible values per symbol. These QAM symbols can be represented using a constellation diagram, which shows the relationship between the symbol and the ASK and PSK components of the signal [28]. The said diagram uses *I* and *Q* to represent the in-phase and quadrature components of QAM-symbols, which depict the phase and amplitude modulation, respectively [29].

The VST is configured to transmit 100,000 known QAM data symbols with a symbol rate of 100 kHz, and to receive the signal after it has passed through the Li-ion cell. These known data begin with training symbols that are used by the VST to perform normalisation and the phase compensation required for effective symbol trace demodulation. The RF output of the VST is connected to the negative terminal of the Li-ion cell, and the positive terminal is connected to the RF input of the VST. The VST and the cell holder are connected via coaxial SMA cables rated to 12.4 GHz at a defined torque. The signal received is demodulated and the received data symbols are then compared with the symbols transmitted. Comparison of the received data with the data transmitted enables calculation of the bit error ratio (BER) and the symbol error ratio (SER), which are ratio-metric comparisons of the corrupted bits and symbols within the data stream, respectively. Since each symbol contains multiple bits, it is observed that the SER will be equal to or greater than the BER for each test.

In addition to measuring BER and SER, the EVM is also calculated in each test, where EVM is a measurement of imperfections within a constellation plot against a reference symbol map. The calculation of the root mean square (RMS) EVM is given by

(4) $${\mathrm{EVM}}_{\mathrm{RMS}}\left(\mathrm{dB}\right)=10\log_{10}\left(\frac{\frac{1}{N}\sum_{k=1}^{N}\sqrt{{\left(I_{k}-{\tilde{I}}_{k}\right)}^{2}+{\left(Q_{k}-{\tilde{Q}}_{k}\right)}^{2}}}{m_{\mathrm{avg}}}\right)$$

where *k* is the symbol index, *N* is the number of symbols, $I_{k}$ is the in-phase measurement of the *k*th symbol, $Q_{k}$ is the quadrature measurement of the *k*th symbol, and $m_{\mathrm{avg}}$ is the average constellation magnitude. ${\tilde{I}}_{k}$ and ${\tilde{Q}}_{k}$ represent measured symbols, whereas $I_{k}$ and $Q_{k}$ represent reference symbols.

**Figure 4:**
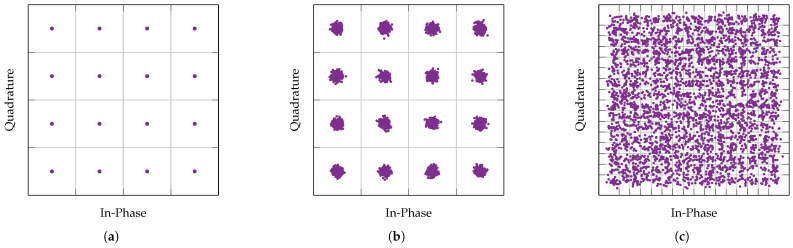
Constellation diagrams showing a reference 16-QAM constellation, and QAM signals with manually added noise. The same number of QAM symbols are tested in each figure. The grid lines depict the relative constellation sector boundaries. (**a**) 16-QAM reference constellation. (**b**) 16-QAM signal with an EVM and BER/SER of −26.4 dB, and 0, respectively. (**c**) 256-QAM signal with an EVM and BER/SER of −25.6 dB, and 0.10, respectively.

The EVM increases as a symbol drifts further away from the reference symbol within a constellation plot. Such imperfections consist of poor phase compensation and normalisation of the VST, which may be caused by significant phase distortion and attenuation by the Li-ion cell. Using EVM enables the performance measurement of the PLC channel in more precise detail, including in instances where the BER or SER do not show any important change. This behaviour occurs because of imperfections in received symbol data may not be significant for the demodulator to corrupt data, but it may be significant to become a cause for concern. These are instances whereby a QAM symbol may be distorted enough to approach another reference symbol, and yet remain sufficiently separated so that the symbol is not demodulated incorrectly. Figure 4 displays a reference 16-QAM constellation diagram with a perfect EVM, and an example constellation diagram of a noisier 16-QAM signal with an RMS EVM of −26.4 dB, respectively.

Higher orders of QAM offer higher data rate as more bits are transferred in each symbol. However, higher orders of QAM require an increased number of symbols on the constellation diagram, which consequently increases the susceptibility of data corruption due to noise. This potential corruption occurs because the QAM order is limited by the signal-to-noise ratio (SNR) of the PLC channel, which must be greater with increasing QAM order to differentiate between different QAM symbols on the constellation diagram [30]. For instance, it can be observed in the 256-QAM signal shown in Figure 4c, that artificially added AWGN noise has a significant negative effect on the PLC system, resulting in increased EVM, bit and symbol errors, as symbols can easily move beyond their correct constellation sector.

To measure the relationship between EVM and the BER and SER, VST output powers of −9 dBm and −27 dBm are tested. The experimental processes of the VST are repeated for each output power selected. The maximum output power of −9 dBm is selected as it remains below the level at which the VST warns of overloading the input RF terminal after the RF signal has passed through the Li-ion cell, which may cause waveform clipping, signal corruption, and potential damage to the equipment. The much lower output power of −27 dBm is expected to cause a great increase in EVM, and highlight further susceptibility to changes in the PLC channel of the Li-ion cell under the test.

**Figure 5:**
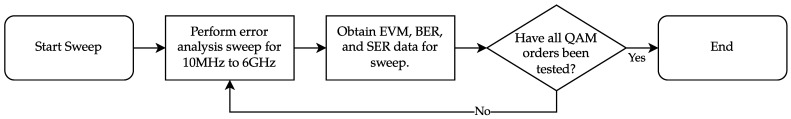
Flowchart of the experimental process specific to the VST. The output power is configured before commencing the sweep.

A summary of the experimental process using the VST is shown in Figure 5.

### 2.3. Cell Holder

To improve the accuracy of all characterisation measurements, the construction and use of the measurement setup must be given careful consideration. The use of winding and unshielded signal paths, such as through a spring or an unshielded cable, may cause irreproducible changes in inductance and capacitance, and thereby complicate the analysis of the measured signals. Existing research has demonstrated the benefits of using a bespoke cell holder for S21 measurements [25].

**Figure 6:**
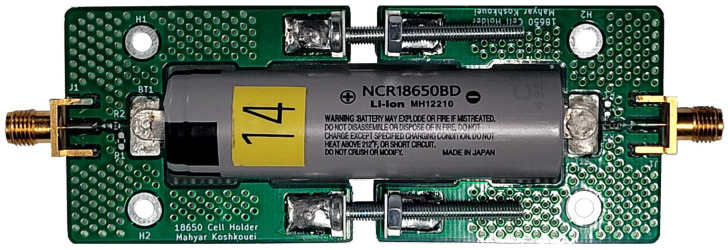
Photo of the Li-ion cell clamped within the cell holder.

Thus, a bespoke cell holder is designed and constructed for particular use within this experiment, and is shown in Figure 6. This cell holder is constructed of two separate printed circuit boards (PCBs) that form a clamp using four screws—two above and two below. A tab in the centre of each cell holder part provides a connection to the terminals of the Li-ion cell. The screws of the cell holder are tightened such that the tabs form a secure connection with the cell terminals, and that the cell is tightly secured within the clamp. Care is taken to ensure that the screws are not over-tightened, which may cause damage to the cell terminals. The Li-ion cell remains clamped within the cell holder under a fixed torque for the full duration of the experiment. Each tab is connected to a signal path that optionally leads through resistors and then to an SMA connector. Both sides of the clamp use a 0 Ω resistor in series to create a bridged connection. This component has been added to the cell holder for the option of using capacitive coupling on either the input or the output of the cell by replacement with a similar-sized ceramic capacitor. However, capacitive coupling is not utilised on the cell holder in this experiment, but alternatively the AC coupling of the input to the VNA and VST is utilised instead. Despite the small size of the resistors selected and hence their low power rating, it is not expected that they have a noticeable effect on the S21 measurements. Since both the VNA and the VST use a high impedance input, the power draw from the cell should be minimal. Therefore, the current passing through the resistors is expected to be very low. The resistors selected are thin-film resistors, which offer negligible effects on the overall circuit’s impedance. As explained in Section 2.1, the characteristic impedance of the cell holder is not considered within this experiment and its effects are removed by using a baseline measurement at maximum SoC, that allows only studying changes of the S21 transmission coefficient with cell SoC. The signal path is as short as possible to reduce the effect of electromagnetic interference (EMI) on the signal. To this end, further techniques include the rounding of footprint edges and using large ground planes on both sides of the PCB that are connected using via stitching. In addition, all screw holes are grounded for further mitigation of EMI on the cell holder.

### 2.4. Lithium-Ion Cell

A single 18650-type Panasonic NCR18650BD-model Li-ion cell is tested in this experiment. The Li-ion cell is tested at SoCs of 95%, 80%, 60%, 40%, 20%, and 5%. The 5% and 95% represent SoCs where the Li-ion cell is near the state of complete discharge and fully charged, respectively. The other SoCs selected are equally separated with a step of 20%. The voltages used for charging and discharging the Li-ion cell to specific SoCs are derived from the data provided on the manufacturer datasheet. The Li-ion cell is to be within ±2% of the desired SoC before commencing each experiment.

The Li-ion cell is discharged to the required SoC using a power resistor and a digital voltmeter for SoC monitoring and automatic safety shut-off. As already stated in Section 2.3, the cell will remain clamped within the cell holder during the discharging process. Once the discharging is completed, the cell holder is disconnected from the discharging apparatus and left to cool-down for a period of 30 min. Based on this experiment, it is observed that the magnitude and phase of the S21 transmission coefficient of the Li-ion cell do not change anymore with time after this cool-down period. The cool-down is essential in maintaining accurate results, as the charging of the Li-ion cell may cause some changes in cell characteristics, such as internal resistance, because of the changes in its core temperature, as described in Section 1.2.

### 2.5. Experimental Procedure

**Figure 7:**
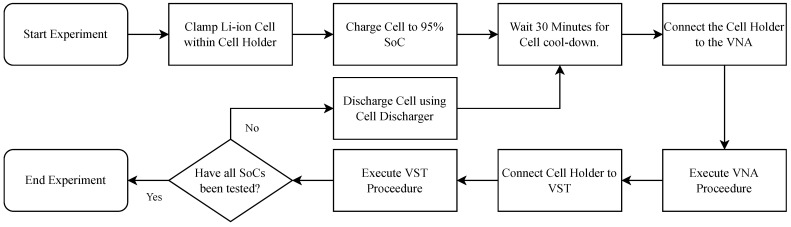
Flowchart of the overall experimental process for obtaining insights into the characteristics of a Li-ion cell at various SoCs and into the associated quality of in situ PLC.

The individual measurement processes of using the Li-ion cell with the VNA and the VST are combined into a single experimental procedure, of which an overview is shown in Figure 7.

To reduce the effects of EMI, the experiment is conducted in WMG’s vehicle drive-in anechoic chamber inside the National Automotive Innovation Centre at the University of Warwick, UK.

The procedure of the experiments is as follows. The Li-ion cell is first prepared by placing it within the cell holder. The screws of the cell holder are tightened enough to keep the cell firmly clamped. The cell is charged to an SoC of 95%. After the cool-down period of 30 min, the cell holder is connected to the VNA. At the second step, with the cell holder connected to the VNA, 100 frequency sweeps are remotely executed consecutively. Thereafter, the cell holder is disconnected from the VNA and connected to the VST. The bespoke program made for this experiment is started on the VST, which executes the transmission and receiving of QAM signal data, and records the received demodulated symbol data and phase error traces for each carrier frequency tested. Finally, the cell holder is disconnected from the VST, and connected to the discharging apparatus for discharging to the next SoC. The process is repeated for all of the SoCs under consideration within the experiment.

The magnitude and phase of the S21 transmission coefficient are retrieved from the VNA and processed to achieve phase compensation, as stated in Section 2.1. These results are combined with the results of other SoCs to highlight the alterations in the transmission coefficient’s magnitude and phase with SoC. The symbol data and phase error traces from the VST are retrieved with the symbol data processed to obtain the EVM, BER, and SER for all SoCs tested. The phase error traces allow for further analysis and investigation into how phase error in QAM symbols may cause instances of increased bit and symbol error.

## 3. Results and Discussion

This section presents the results obtained from the experimentation described in Section 2, and discusses the changes in cell characteristics with SoC by considering PLC performance.

Using the S21 magnitude and phase shift data of the 100 sweeps performed, and taking into account the standard deviation within such single set of measurements and the reproducibility in cell charging, the achieved error margins are ±0.1 dB and ±1.0 dB, respectively.

### 3.1. S21 Analysis

**Figure 8:**
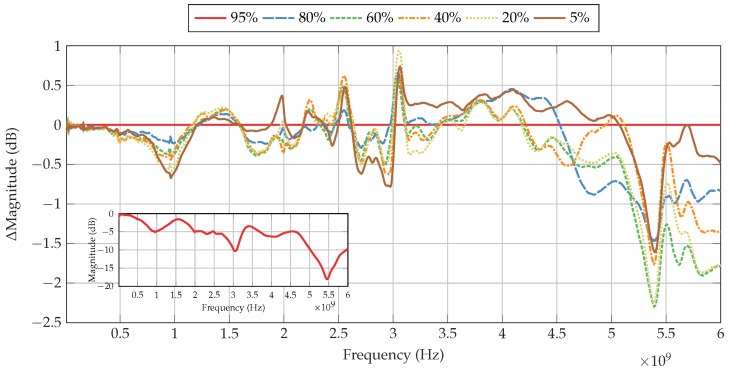
Change in S21 magnitude of the Li-ion cell at various SoCs, using 95% SoC as a baseline. Inset shows the baseline S21 magnitude of the Li-ion cell at 95% SoC.

Figure 8 shows the change in S21 magnitude of the Li-ion cell as the SoC is decreased in comparison to the baseline of 95% SoC, presented in the inset. It can be observed that the change in S21 magnitude is dependent on the frequency tested. Furthermore, some frequencies display greater change with SoC than others.

The first major deviation in S21 magnitude can be observed at 964 MHz, whereby the S21 magnitude of the signal across the Li-ion cell at 5% SoC is decreased by 0.7 dB in comparison to the baseline of 95% SoC. In addition, between frequencies of 904 MHz and 975 MHz, it can also be observed that there is a gradual, almost linear trend between the S21 magnitude of the signal and the SoC, whereby the S21 magnitude is reduced with decreasing SoC, as presented in Table 1.

This trend is broken as the frequency rises from 975 MHz, whereby the change in magnitude with SoC decreases to becoming SoC independent in the frequency range from 1157 MHz and 1225 MHz. At 1982 MHz, a minor peak of 0.4 dB can be observed with 5% SoC only, with the other SoCs remaining with a negative magnitude relative to the baseline. As the frequency increases, the change in magnitude with SoC rises and falls between ±0.5 dB with no clear trend visible. At 2549 MHz, a peak of 0.6 dB can be observed with 40% SoC. Peaks of smaller change in magnitude can also be observed with the other SoCs, where with 80% SoC having the smallest change in magnitude relative to the baseline.

This peak in magnitude sharply falls to a broad negative peak from 2592 MHz to 3018 MHz. Within this range, a weak trend can be observed between 2662 MHz and 3009 MHz, whereby 5% SoC shows the lowest magnitude relative to the baseline. This trend is briefly lost between 2757 MHz and 2867 MHz, where 20% to 80% SoC are less than −0.2 dB and within 0.1 dB of each other, whilst 5% SoC remains more than −0.4 dB from the baseline. A sharp peak can be observed at 3052 MHz, which shows the greatest positive difference in magnitude of 0.9 dB relative to the baseline for all frequencies tested. After this peak, the change in magnitude falls and remains within ±0.5 dB until 4500 MHz where the magnitudes begin to decrease relative to the baseline. This decrease continues until a peak is reached at 5397 MHz of −2.3 dB for 60% SoC. At this peak, whilst 80% SoC displays the lowest change in magnitude relative to the baseline compared to the other SoCs, it is the greatest change in magnitude from the baseline for this SoC for all frequencies tested. After this peak, the magnitudes of each SoC remain the furthest apart up to 6000 MHz, with 5% SoC approaching the baseline, whereas the other SoCs are spread relatively evenly up to −1.9 dB.

### 3.2. Phase Analysis

**Figure 9:**
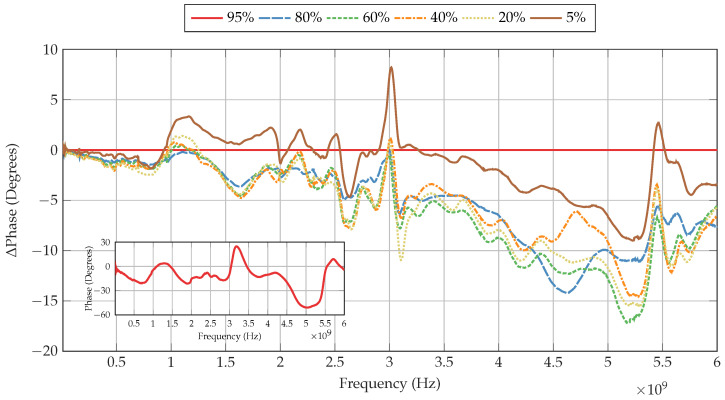
Change in phase of a Li-ion cell at various SoCs, using 95% SoC as a baseline. Inset shows the baseline phase shift of the Li-ion cell at 95% SoC with the background removed.

Figure 9 presents the change in phase of the Li-ion cell as the SoC is decreased in comparison to the baseline of 95% SoC, shown in the inset. Such as the changes in S21 magnitude described in Section 3.1, it can be observed that the phase also changes with the tested carrier frequencies. Some sharp changes in phase occur at the same frequencies where sharp changes in S21 magnitude take place; however, there are some distinct differences, particularly for 5% SoC, as shown in Table 2.

As the frequency increases from 10 MHz, the phase shifts for all SoCs decrease, and follow a weak negative trend, whereby lower SoCs display a greater phase shift when 5% SoC is not considered. The lowest SoC of 5% yields the smallest phase shift, whilst the phase shift of 20% remains farthest from the baseline. This weak trend continues until 875 MHz, where the phase shifts of all SoCs begin to decrease by varying amounts. From 936 MHz, the phase shift of 5% SoC rises, reaching a peak of 3.3${}^{{}^{\circ}}$ at 1165 MHz. The phase shifts of the other SoCs also rise, but by a much lower amount. In fact, only the phase shift of 80% SoC remains negative at this frequency with a value of −0.2${}^{{}^{\circ}}$. The difference in relative phase shift between 5% SoC and the other SoCs remains intact. This continues through the minimum in phase at 1649 MHz, whereby 40% SoC displays the highest relative phase shift of −4.8${}^{{}^{\circ}}$, followed closely by 60%, 20% and 80% SoC. The relatively large phase shift of 5% SoC begins to subside at 1925 MHz and approaches the other SoCs at 2001 MHz. From this frequency, the phase shift of 5% SoC remains up to 7.5${}^{{}^{\circ}}$ from any other SoC apart from the baseline. Despite this behaviour, the same features that are present with the other SoCs are also displayed in 5% SoC. This includes the sharp minimum at 2632 MHz, whereby 5% SoC reaches a relative phase shift of −4.7${}^{{}^{\circ}}$, whereas the largest phase shift recorded at this frequency is −7.8${}^{{}^{\circ}}$ with 40% SoC. At this frequency, the phase shift of 80% SoC is shown to deviate from the SoCs of 20%, 40% and 60% where at lower frequencies these SoCs are within 1${}^{{}^{\circ}}$ from each other; the phase shift of 80% SoC is now the same as that for 5% SoC.

Beginning at 2907 MHz, a large maximum develops. At 3015 MHz, 5% SoC produces the greatest rise in phase shift at this frequency, reaching a peak of 8.2${}^{{}^{\circ}}$. The same feature can be observed in the other SoCs, whereby the positive change in phase shift causes the relative negative phase shift of these SoCs to approach the baseline. After this peak, the phase shifts of all SoCs begin to fall, with the phase shift of 5% SoC approaching the baseline, and the other SoCs becoming more negative, with 20% SoC reaching −10.9${}^{{}^{\circ}}$ at 3106 MHz.

As the frequency increases, the relative phase shifts of all SoCs increase. Between 3180 MHz and 4000 MHz minor peaks exist in SoCs of 5%, 20%, 40% and 60%. In contrast, 80% SoC does not display such features, but rather gradually increases its phase shift, similar to the other SoCs, and continues to do so from 4350 MHz. Where the phase shifts of the other SoCs cease to increase here, the phase shift of 80% SoC reaches a local maximum of −14.2${}^{{}^{\circ}}$. Afterwards, the phase shift of 40% SoC decreases, approaching the phase shift of 5% SoC, and reaching a local minimum of −6.1${}^{{}^{\circ}}$. Despite this behaviour, the phase shift of 40% SoC rapidly increases and approaches 20%, 40% and 60% SoC, whilst the phase shift of 80% SoC decreases. The largest maximum of −17.2${}^{{}^{\circ}}$ develops at 5187 MHz with 60% SoC. Following this, a sharp decrease in absolute phase shift occurs, with a local minimum of −3.5${}^{{}^{\circ}}$ at 5450 MHz with 40% SoC, and the phase shift of 5% SoC becoming positive. After this sharp peak, the phase shifts of all SoCs gradually decrease until 6000 MHz, where the maximum relative phase shift is −7.6${}^{{}^{\circ}}$ with 80% SoC.

Some features of relative phase shift discussed can be observed in all of the SoCs. It may thus be deduced that the actual phase shift of the 95% SoC baseline may have exhibited such features such as the sharp peak at 3015 MHz, which leads to proportional changes in the phase shift of the other SoCs tested.

### 3.3. Communication Error Analysis

**Figure A1:**
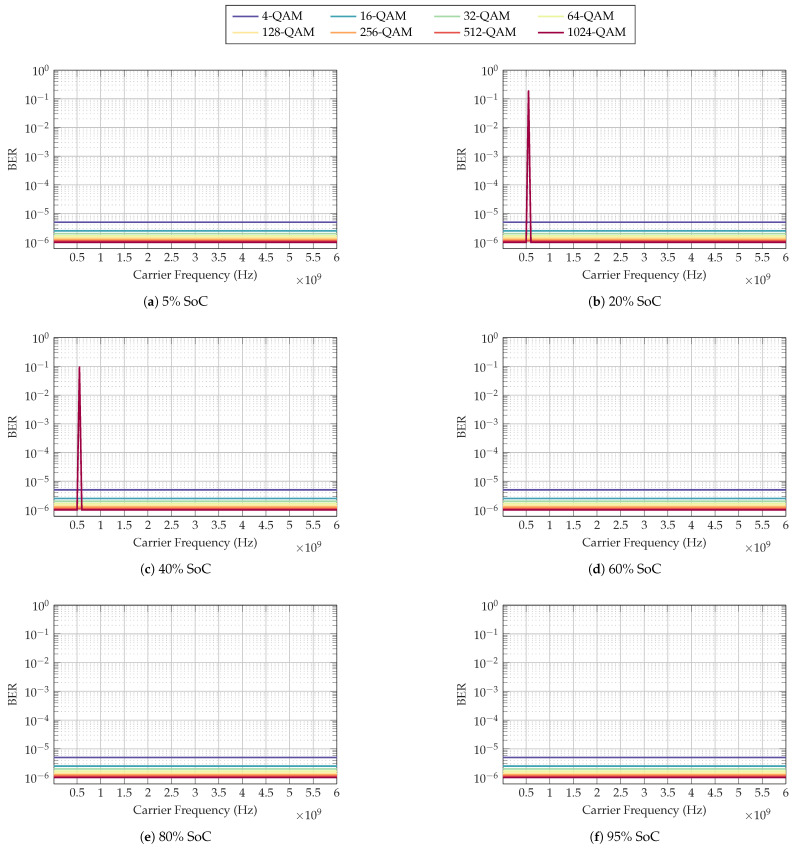
BER for QAM-signals transmitted with a peak output power of −9 dBm through a Li-ion cell at various SoC. Each QAM order is represented in a different colour. Each order of QAM has a different minimum BER since the number of transmitted symbols is kept constant, rather than the number of transmitted bits.

**Figure A2:**
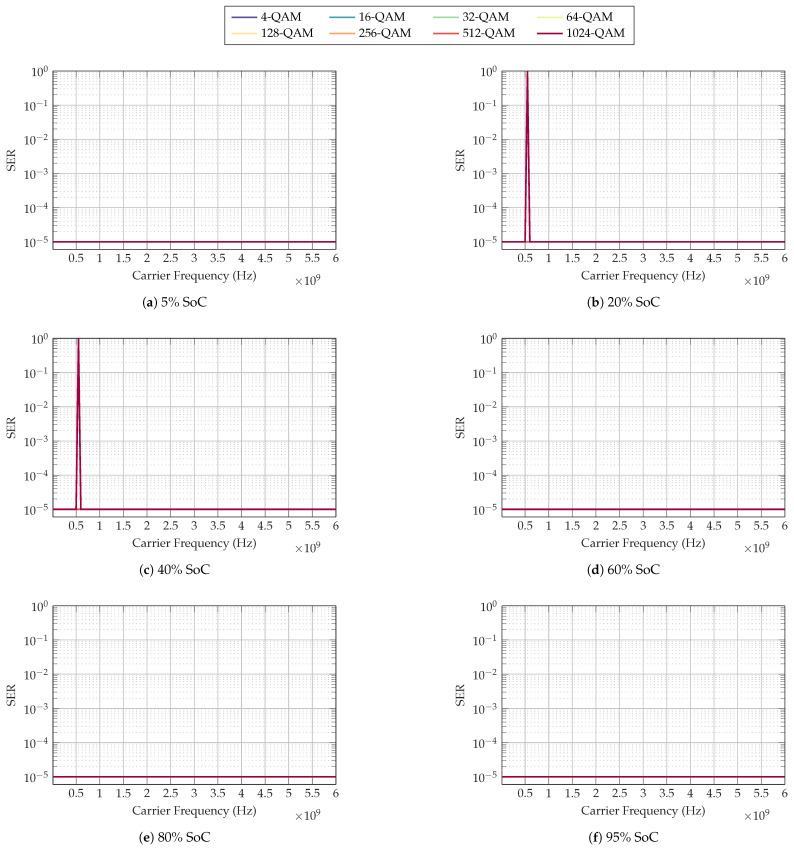
SER for QAM-signals transmitted with a peak output power of −9 dBm through a Li-ion cell at various SoC. Each QAM order is represented in a different colour.

In this section, the BER, SER and EVM of the PLC system are analysed and compared. The full results for BER and SER are presented in the Appendix A in Figure A1 and Figure A2, respectively. The results are the same for both −9 dBm and −27 dBm output power, hence the results for −27 dBm are omitted for brevity. These results indicate that there is no significant attenuation or phase shift on the PLC channel that results in any bit or symbol errors. However, the VST performs automatic filtering of the input RF signal, including normalisation and phase compensation. These two techniques are typically required for QAM demodulators, as they allow for correct mapping of QAM symbols to the constellation space [31].

**Figure 10:**
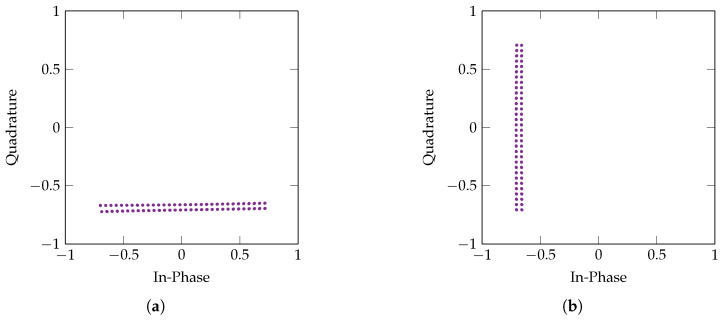
Scatter plots of the first 100 QAM symbols received by the VST during 1024-QAM PLC. (**a**) Carrier frequency of 550 MHz. (**b**) Carrier frequency of 600 MHz.

The normalisation and phase compensation performed on the input RF signal removes all but a single increase in BER and SER at 550 MHz with 20% and 40% SoCs. At this frequency, the automatic phase shift compensation fails for the highest modulation order of 1024-QAM. Figure 10 displays the scatter plots of the first 100 symbols during a test of 1024-QAM at 550 MHz and 600 MHz, respectively. While 600 MHz shows the lowest error ratio possible, the constellation of the 550 MHz symbol trace is phase shifted by −90${}^{{}^{\circ}}$, which has caused the maximum in BER and SER.

**Figure 11:**
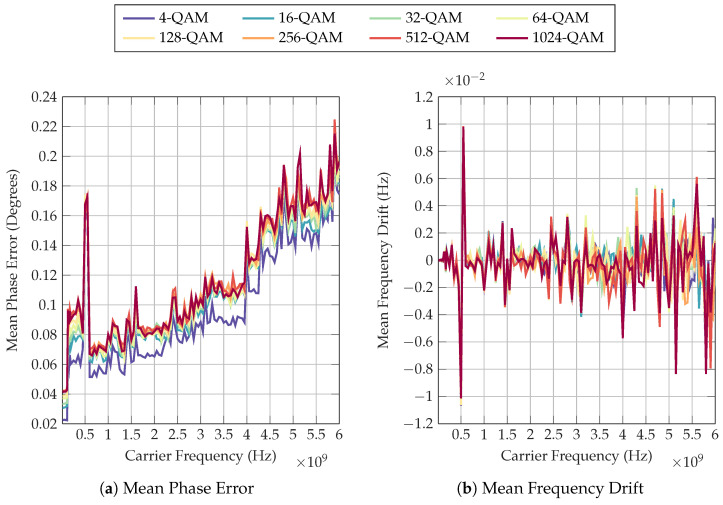
Mean phase error and mean frequency drift as detected by the VST for the 20% SoC test.

**Figure A3:**
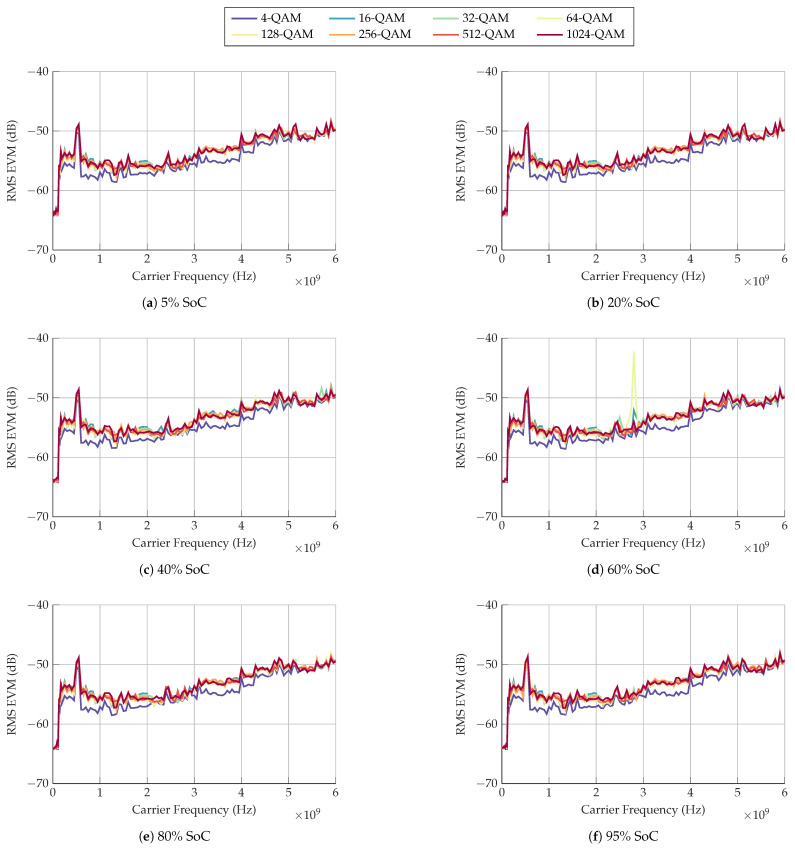
RMS EVM for QAM-signals transmitted with a peak output power of −9 dBm through a Li-ion cell at various SoC. Each QAM order is represented in a different colour.

**Figure A4:**
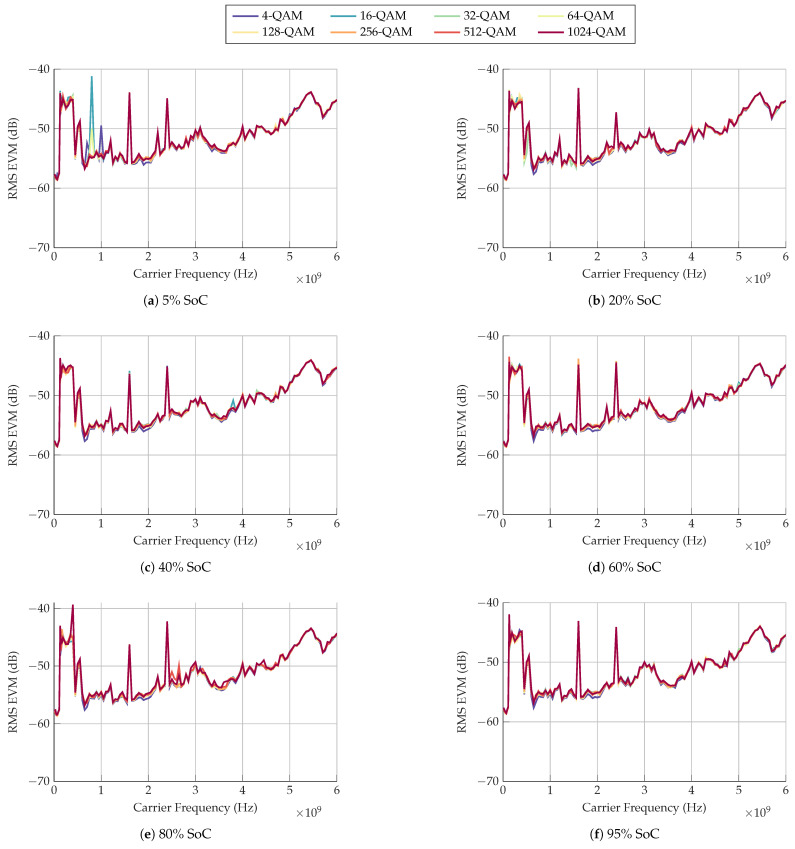
RMS EVM for QAM-signals transmitted with a peak output power of −27 dBm through a Li-ion cell at various SoC.

In Figure 11 at 550 MHz, a slight increase in mean phase error and frequency drift can be observed. The definitions of mean phase error and frequency drift can be found in [28]. These increases in error and drift can be associated with the observation of incorrect phase shift compensation shown in Figure 10. Furthermore, EVM results presented in Figure A3 and Figure A4 all display a large increase in EVM at 550 MHz, which remains constant regardless of the output power. Conversely, no significant peak can be observed at 550 MHz in S21 magnitude or phase shift, as presented in Section 3.1 and Section 3.2. It can therefore be hypothesised that this error is not likely due to a change in characteristics of the Li-ion cell, but rather caused by an external factor, including the signal path and filter configuration used within the VST causing higher error ratios at this specific frequency.

The EVM results for −9 dBm and −27 dBm output power are presented in the Appendix A in Figure A3 and Figure A4, respectively. It can be observed that the reduced output power causes a slight rise in EVM for all carrier frequencies tested. Some carrier frequencies display increased levels of EVM when an output power of −27 dBm is used. The first large increase is from 130 MHz to 400 MHz, whereby the EVM rises from −58 dB to −44 dB for all SoCs. In Figure A4e a peak in EVM of 1024-QAM occurs, reaching a maximum of −39 dB, before returning to the same EVM levels observed with other SoCs. There is no noticeable correlation between this wide peak in EVM with the S21 magnitude and phase results determined with the VNA. Another peak in EVM occurs at 800 MHz for 5% SoC, as shown in Figure A4a, for 4, 32, 64 and mostly for 16-QAM, which reaches a value of −41.2 dB.

Another peak specific to 5% SoC is observed at 1000 MHz, whereby the EVM of only 4-QAM rises to −49.5 dB. This peak may also be attributed to the sudden alteration in S21 magnitude and phase shift, where 5% SoC had shown changes distinct from the other SoCs tested. Since only 4-QAM shows this increase in EVM, it may be deduced that the VNA is unable to normalise or phase-compensate the 4-QAM symbol trace as effectively as the other modulation orders tested.

**Figure 12:**
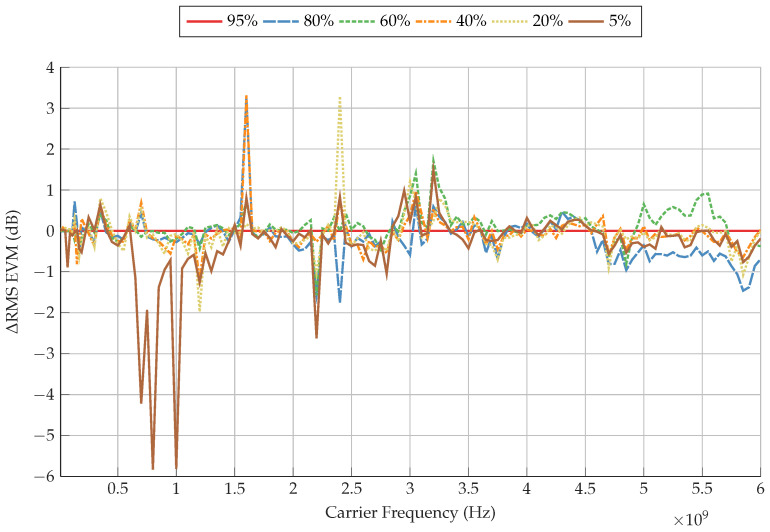
Change in RMS EVM of 4-QAM with SoC, using 95% SoC as a baseline.

Figure 12 clarifies how significant the changes in EVM are at the frequencies of 700 MHz, 800 MHz and 900 MHz for 5% SoC, which reach −4.2 dB, −5.8 dB and −5.8 dB relative to the baseline of 95% SoC, respectively. Because of the reduced step in frequency used on the VST in comparison to the VNA as explained in Section 2.2, the peak in EVM at these specific frequencies is not recorded. Hence, the specific cause of this increased level of EVM is not conclusive as to whether it is due to the peak in phase, peak in S21 magnitude, or a combination of both. It should be noted that the normalisation and phase compensation process of the VNA is unable to completely filter this peak in EVM for these modulation orders, but is able to do this for 128, 256, 512, and 1024-QAM.

The next peak in EVM occurs at 1600 MHz for all SoCs, reaching an absolute value of −43.0 dB. Figure 12 shows that relative to 95% SoC, 40% SoC has the greatest EVM at this peak, whereas 95% SoC has the lowest EVM. At this frequency, a correlation can be observed with the gradual minima occurring in S21 magnitude and a peak in negative phase shift, relative to their baselines.

A smaller peak in EVM occurs at 2200 MHz, reaching to an EVM of −50.6 dB with 5% SoC. At this frequency, Figure 12 shows that there is a variation of up to −2.6 dB in EVM, where 5% SoC has the highest EVM and 95% SoC has the least. Furthermore, the largest peak in EVM can be observed at 2400 MHz, reaching −42.2 dB with 80% SoC. A large variation in EVM occurs at this frequency, with 20% SoC showing the least EVM of −47.3 dB. As has been shown before, small peaks in both S21 magnitude and phase also occur at these frequencies.

Commencing 3500 MHz, the EVM for all SoCs tested begins to rise gradually up to 6000 MHz. At 4700 MHz, the differences in EVM between the SoCs tested begin to diverge to a maximum of 1.5 dB. A broad minimum can be observed at 5700 MHz, which decreases the EVM in all SoCs. A similar characteristic can be observed in both S21 magnitude and phase results, which display a slight decrease in S21 magnitude and a significant decrease in negative phase shift.

### 3.4. Overall Analysis and Discussion

The presented experimental results have demonstrated that changes in Li-ion cell SoC cause changes in in situ PLC channel characteristics of both the S21 magnitude and phase over the RF frequency range from 10 MHz to 6 GHz. At some frequencies, sharp changes in these characteristics occur, which cause an increase in EVM. Exemplary frequencies include sharp changes in S21 magnitude and phase at 1165 MHz and 3015 MHz, respectively, which occur for all SoCs tested relative to the respective baselines of 95% SoC. In addition, it is demonstrated that 5% SoC yields the largest change in phase shift for most frequencies, whereas the largest change in magnitude is not observable with 5% SoC. In fact, 60% SoC displays the largest change in magnitude for all frequencies tested at 5397 MHz. At certain frequencies, some trends between the SoC and both the S21 magnitude and phase are noticed, whereby the S21 magnitude and phase characteristics are in an almost linear order of 95% to 5% SoC. This behaviour can be observed between frequencies of 904 MHz and 975 MHz for S21 magnitude, and a weaker trend at 10 MHz and 875 MHz for phase shift, where only the phase shift of 5% SoC deviates from this trend by remaining consistently between 95% and 80% SoC.

The EVM results verify that some increases in error can be attributed to certain changes in the characteristics of the Li-ion cell. In particular, it is found that large changes in S21 magnitude and phase correlate to specific increases in EVM, such as for carrier frequencies between 1600 MHz and 2400 MHz. However, BER and SER results indicate that these increases in EVM are not significant to impede the successful demodulation of the signal data by the VST. The VST uses a filtered signal path, with normalisation and phase shift compensation, which is expected to automatically improve the results. Nevertheless, these are features that are available to most QAM demodulators, as explained in Section 2.

Based on this study, it can be implied that smaller changes in S21 magnitude and phase with SoC at a given frequency will benefit an in situ Li-ion PLC network. This is because less reconfiguration of the transmitter and the receiver will be required as the Li-ion cell’s SoC changes. For example, if 3000 MHz is selected as a PLC carrier frequency, it is possible that data corruption could occur when the SoC of the Li-ion cell is decreased to 5% due to the relatively large changes that occur in S21 magnitude and phase shift of 0.9 dB and 8.2${}^{{}^{\circ}}$, respectively.

From the results presented in this study, the appropriate carrier frequencies for an in situ Li-ion PLC network with consideration of the SoC of the Li-ion cell, can be established. Firstly, the frequency range from 50 MHz to 400 MHz displays little variation in S21 magnitude and phase shift with SoC of 0.1 dB and 1.7${}^{{}^{\circ}}$, respectively. This variation in S21 magnitude and phase shift increases marginally up to 800 MHz, whereas significant changes in EVM begin to occur at 600 MHz with multiple peaks present up to 1050 MHz. In addition, between 1133 MHz and 1652 MHz, the difference in S21 magnitude and phase shift between SoCs is insignificant, remaining at less than 0.2 dB and 4.8${}^{{}^{\circ}}$, respectively. Further carrier frequencies of recommendation include the range from 3300 MHz to 4000 MHz, which illustrates low variation in S21 magnitude and phase shift of the Li-ion cell with SoC of a maximum of 0.4 dB and −11.6${}^{{}^{\circ}}$, respectively.

Because of commonly used normalisation and phase compensation techniques already established within QAM demodulation, it is unlikely that an increase in QAM order will have a negative effect on the PLC system’s performance. Nevertheless, the limitations of the QAM signal filtering must be considered before increasing QAM order. The presented BER and SER data demonstrate that the VST is unable to correctly phase compensate a 1024-QAM symbol trace. It is therefore appropriate to select a lower modulation order QAM to ensure a higher immunity to external factors, such as noise.

## 4. Conclusions

In this paper, the changing characteristics of an 18650-model lithium-ion cell with varying states of charge (SoCs) as an in situ power line communication (PLC) channel have been identified. The performance of the in situ Li-ion PLC system has been validated for SoCs of 5%, 20%, 40%, 60%, 80%, and 95% by conducting communication tests with data modulated with quadrature amplitude modulation (QAM) in orders of 4, 16, 32, 64, 128, 256, 512, and 1024-QAM for high throughput communication.

It has been demonstrated that the SoC of the Li-ion cell has an impact on the PLC channel characteristics of the transmission coefficients, S21, magnitude, and phase. These changes in SoC strongly depend on the used carrier frequency. In fact, it was found that the Li-ion cell at 5% SoC had the most significant negative impact on both the S21 magnitude and phase, occurring at 1165 MHz, 3015 MHz, and 5450 MHz. At these frequencies, significant changes in both S21 magnitude and phase shift are observed, reaching a maxima of 2.4 dB and 19.9${}^{{}^{\circ}}$ with respect to the baseline, respectively. Based upon empirical evidence presented within this paper, it can be concluded that the most appropriate parameters for an in situ Li-ion PLC system that utilises QAM should be selected as follows: the selection of a carrier frequency between 50 MHz to 400 MHz, 1133 MHz to 1652 MHz, or 3300 MHz to 4000 MHz; using a maximum modulation order of 512-QAM; and the use of normalisation and phase compensation within the QAM demodulator.

In these experiments, the effects of reduced transmission power have shown an increase in error vector magnitude. This consequently requires the selection of a lower modulation order of QAM for improved resistance to changes in Li-ion cell characteristics with SoC and to external factors such as noise. Such noisy environments require the use of a lower modulation order of QAM.

Further research is ongoing to determine the effects of Li-ion cell state of health on an in situ PLC system, and the differences in PLC channel characteristics between Li-ion cells of a different model and manufacturer.

## Figures and Tables

**Table 1:** Change in S21 magnitude (in dB) with SoC at selected frequencies using a baseline of 95% SoC.

	SoC	5%	20%	40%	60%	80%
Frequency						
964 MHz		−0.7 dB	−0.6 dB	−0.5 dB	−0.4 dB	−0.2 dB
1982 MHz		0.4 dB	0.0 dB	−0.3 dB	−0.2 dB	−0.1 dB
2549 MHz		0.5 dB	0.4 dB	0.6 dB	0.5 dB	0.2 dB
2709 MHz		−0.6 dB	−0.5 dB	−0.5 dB	−0.4 dB	−0.3 dB
2978 MHz		−0.8 dB	−0.3 dB	−0.6 dB	−0.2 dB	−0.0 dB
3052 MHz		0.7 dB	0.9 dB	0.6 dB	0.5 dB	0.6 dB
5397 MHz		−1.6 dB	−2.2 dB	−1.7 dB	−2.3 dB	−1.5 dB
6000 MHz		−0.5 dB	−1.8 dB	−1.4 dB	−1.8 dB	−0.8 dB

**Table 2:** Change in phase shift (in degrees) with SoC at select frequencies using a baseline of 95% SoC.

	SoC	5%	20%	40%	60%	80%
Frequency						
820 MHz		−1.6°	−2.2°	−1.8°	−1.6°	−1.5°
1165 MHz		3.6°	1.4°	0.1°	0.2°	−0.3°
1649 MHz		1.0°	−4.1°	−4.8°	−4.4°	−3.7°
2001 MHz		−1.1°	−2.8°	−2.2°	−2.2°	−2.7°
2632 MHz		−4.4°	−7.2°	−7.8°	−6.9°	−4.6°
3015 MHz		8.4°	0.5°	1.0°	−1.4°	−0.7°
3106 MHz		0.5°	−10.5°	−6.7°	−7.5°	−6.0°
5187 MHz		−8.6°	−15.2°	−14.3°	−17.1°	−11°
5450 MHz		2.6°	−3.7°	−3.6°	−6.8°	−5.6°
6000 MHz		−3.2°	−5.4°	−6.5°	−5.6°	−7.6°

## Data Availability

The data presented in this study are available at https://wrap.warwick.ac.uk/165898, (accessed on 29 July 2022) under the terms of the CC-BY license. Due to the large file size, extensive data is available from the corresponding author upon reasonable request.

## Abbreviations

The following abbreviations are used in this manuscript:

ASK	Amplitude Shift Keying
BER	Bit Error Ratio
BEV	Battery Electric Vehicle
BMS	Battery Management System
CAN	Controller Area Network
EMI	Electromagnetic Interference
EVM	Error Vector Magnitude
PCB	Printed Circuit Board
PLC	Power Line Communication
PSK	Phase Shift Keying
QAM	Quadrature Amplitude Modulation
RMS	Root Mean Square
SER	Symbol Error Ratio
SNR	Signal to Noise Ratio
SoC	State of Charge
SoH	State of Health
VNA	Vector Network Analyser
VST	Vector Signal Transceiver
